# *Salmonella* serovars in sheep and goats and their probable zoonotic potential to humans in Suez Canal Area, Egypt

**DOI:** 10.1186/s13028-022-00637-y

**Published:** 2022-07-29

**Authors:** Hanan Abd El-Halim Hawwas, Abdel-Karim Mahmoud Aboueisha, Hanaa Mohamed Fadel, Heba Sayed El-Mahallawy

**Affiliations:** Department of Hygiene, Zoonoses and Animal Behaviour, Faculty of Veterinary Medicine, Suez Canal University, 4.5 Kilo Ring Road St., Ismailia, 41522 Egypt

**Keywords:** Salmonellosis, Small ruminants, Virulence genes, Zoonoses

## Abstract

**Background:**

*Salmonella* is one of the most common and economically important zoonotic pathogens. This study aimed to determine the occurrence of *Salmonella* serovars in sheep and goats and their probable zoonotic risk to humans in Suez Canal area in Egypt. A total of 320 fecal samples from sheep (n = 120), goats (n = 100), and humans (n = 100) were collected and examined for the presence of *Salmonella* based on cultural and biochemical characteristics, and serological analysis. Moreover, the virulence of the identified *Salmonella* isolates was assessed by molecular screening for *inv*A, *stn*, *spv*C, and *sop*B virulence genes using PCR.

**Results:**

Overall, the occurrence of *Salmonella* in sheep feces (23.3%) was higher than that in goat feces (7%) and human stool (13%) in the study area. The identified isolates belonged to 12 serotypes; ten, five, and eight from sheep, goats, and humans, respectively. The most frequently identified serotypes were *S.* Typhimurium from sheep feces, and *S.* Enteritidis from both goat feces and human stool, with four serotypes; *S.* Typhimurium*, S.* Enteritidis*, S.* Dublin and *S.* Saintpaul, were mutually shared between all of them. Demographic data revealed that diarrheic sheep (85.7%) and goats (25%) had a higher risk for *Salmonella* fecal carriage than non-diarrheic ones (19.5% and 6.25%, respectively). The prevalence of *Salmonella* infection in humans in contact with sheep and goats (28%) was significantly higher than its prevalence in people having a history of contact with animals other than sheep and goats (10%) and those having no history of animal contact (7.3%) (χ^2^ = 6.728, P ˂ 0.05). The *stn*, *spv*C, and *sop*B genes were detected in 98.1% of the isolates, with a significant, very strong positive correlation for their mutual presence (P < 0.05). Approximately 40.7% of isolates that carried the *inv*A gene had a non-significant, very weak positive correlation with other virulence genes. The most common genotypic virulence profile for all isolates was *stn*, *spv*C, and *sop*B; however, *inv*A, *stn*, *spv*C, and *sop*B was the frequent virulotype for *S.* Typhimurium*, S.* Tsevie*, S.* Apeyeme*,* and *S.* Infantis*.*

**Conclusions:**

The present study highlights the role of apparently healthy and diarrheic sheep and goats as reservoirs and sources of human infection with virulent *Salmonella* serovars in the Suez Canal area.

## Background

Salmonellosis is an infectious disease of humans and animals caused by bacteria of the genus *Salmonella*. Globally, nontyphoidal *Salmonella* has been estimated to cause 93.8 million cases of *Salmonella*-induced gastroenteritis each year, with 155,000 deaths occurring as a result [1]. More than 2600 serovars have been recognized to date, with more than 50% belonging to *S*. *enterica* subsp. *enterica*, which is responsible for the vast majority of human salmonellosis cases [2]. Despite being one of the most significant zoonotic food-borne infections in Egypt and the reporting of human infections globally, there are no accurate statistical data on the health and economic impact of food-borne salmonellosis [3].

The annual growth rates of the sheep and goat livestock industry in Egypt are higher than those of all other North African countries [4]. Sheep and goats are the second most consumed farm animal meat after bovines; moreover, they are becoming popular home-raised species in many houses in pre-urban and rural ranges as a source of economy [5]. However, they represent an economic and potential public health risk, as several organisms could be subclinically carried in these animal reservoirs [6]. Due to limited research in Egypt, their role in transmitting zoonotic infections is underestimated.

Generally, *Salmonella* infection in humans ranges from a self-limiting condition to a life-threatening one that causes death in children, old people, and immunodeficient persons [7]. In addition to the commonly known food-borne infection route, humans can become infected through interactions with live animals and environments contaminated with animal fecal matter, followed by the subsequent accidental ingestion of the bacteria [8]. Moreover, animal gut microbiota and associated pathogens in the intestines or feces of animals may contaminate the carcass and easily be transferred to the flesh, organ surfaces, fleece, and skin during meat processing. Additionally, this may occur indirectly through human hands and equipment in the slaughterhouse [6].

In recent years, the investigation of virulence genes and their roles in pathogenicity mechanisms has been proposed as a method of characterizing *Salmonella* isolates. This includes the detection of representative candidates from the pathogenicity islands such as the *sop*B gene that is shared by all serotypes and is responsible for inducing inflammation and mediating the level of fluid secreted into the gut of an infected host [9], the *inv*A gene that is responsible for the invasion of host epithelial cells by *Salmonella* and is used as a special marker for the quick detection of *Salmonella* [2], and the enterotoxin production *stn* gene that is involved in the complex mechanism of diarrhea due to *Salmonella* infection [10]. Also, the virulence plasmid C (*spv*C) gene is associated with the systemic dissemination of *Salmonella* and the suppression of innate immunity in infected hosts [11].

Generally, there is insufficient regional incidence data on salmonellosis in North Africa [1] and few published studies on *Salmonella* spp. carriage in sheep and goats are available. Few previous studies in Egypt have documented the occurrence of *Salmonella* in diarrheic sheep and goats only, and/or restricted to kids and lambs [12–14]; however, none of them have focused on *Salmonella* prevalence in both diarrheic and apparently healthy sheep and goats and its relationship with *Salmonella* carriage in human contacts. Therefore, this study aimed to determine the occurrence of *Salmonella* serovars and their virulence in sheep and goats in the Ismailia and Port-Said governorates in Egypt and the associated risk to humans in the study area.

## Methods

### Study area

The current study was performed in two governorates of the Suez Canal Area. Port-Said governorate is located in the northeast of Egypt, extending about 30 km along the coast of the Mediterranean Sea, north of the Suez Canal with geographic coordinates Latitude: 31°15′23″ N and Longitude: 32°17′02″E. Ismailia governorate is situated on the west bank of the Suez Canal, approximately halfway between Port-Said to the north and the Suez governorate to the south. The protocol for sample collection and laboratory examination has been reviewed and approved by the Scientific Research Committee and Bioethics Board of the Faculty of Veterinary Medicine, Suez Canal University, Ismailia, Egypt.

### Sample collection and preparation

A total of 320 fecal samples were collected from sheep (n = 120), goats (n = 100), and humans (n = 100) (Table 2). In sheep and goats, samples were collected from various rural and pre-urban localities where household animals were kept in small groups. For clinically healthy animals, fecal samples were collected directly from the rectum using the fingertip after wearing sterile gloves and aseptically placed in labeled sterile capped containers. Additionally, sterile swabs in buffered peptone water were used for the collection of fecal samples from diarrheic animals. The ages and sexes of the animals were recorded and data on the type and source of animal feed and water and farm hygiene were obtained from the owners of the animals who gave their informed consent for the animals to be used in this study.

Human stool samples were collected from patients [including those suffering from diarrhea and fever (diseased) and those admitted for routine fecal examination (clinically healthy)] admitted to general hospitals and private laboratories in the suburbs near animal collection sites in Port-Said and Ismailia governorates. After the patients gave their oral informed consent, data on their ages, sexes, residences, and history of contact with animals were collected under the supervision of private laboratories and hospital administrators.

All samples were transferred immediately in a cold box (4 °C) to the research laboratory for Zoonoses, Faculty of Veterinary Medicine, Suez Canal University. For pelleted animal samples, approximately 10 g of each fecal sample were placed in sterile stomacher bags with 45 mL of buffered peptone water (BPW) (Oxoid, UK) and homogenized in a stomacher (Lab. Blender 400, Seward Lab, London, UK) for 1 min while soft fecal samples were used directly.

### Bacteriological isolation and identification of Salmonella spp.

Isolation was performed according to a previously described protocol (ISO, 2007) (ISO 6579:2002/Amd 1:2007, Annex D). Specimens were pre-enriched in BPW (Oxoid, UK) at 37 ± 1 °C for 18 ± 2 h. From this pre-enrichment broth, a 0.1 mL aliquot was transferred to 9 mL of Rappaport-Vasiliadis (Oxoid, UK) broth and incubated at 41 ± 1 °C for 24 ± 3 h, and another 1-mL aliquot was transferred to 10 mL of Simmons’ Citrate (Oxoid, UK) broth and incubated at 37 ± 1 °C for 18 h for enrichment. Selective plating was then performed by streaking two loopfuls from each enrichment broth onto xylose-lysine-desoxycholate agar (Lab M, UK) plates and incubated at 37 ± 1 °C and examined after 24 ± 3 h. Based on cultural characteristics and morphological features in gram-stained films, presumptive colonies were picked up and processed for biochemical characterization as previously described [15], where oxidase, catalase, TSI, indole production, methyl red-Voges-Proskauer, urease, and citrate utilization were performed. A single pure colony from each isolate was subcultured on nutrient semisolid agar at 37 ± 1 °C for 24 h and preserved for further serological and molecular characterization.

### Serotyping of Salmonella isolates

Fresh pure cultures were sent to the Food Analysis Laboratory of the Faculty of Veterinary Medicine, Benha University, to be identified based on the O “somatic” antigen (using polyvalent O antiserum and individual monovalent O antiserum) and H “flagellar” antigen phase 1 and phase 2 (using polyvalent H antiserum and individual monovalent H antiserum) using commercial kits (Denka Seiken, Japan).

### Molecular detection of virulence genes in Salmonella isolates

DNA from pure isolates was extracted according to the QIAamp DNA mini kit (QIAGEN, Hilden, Germany) instructions. The different virulence genes, *inv*A*, sop*B, *spv*C, and *stn,* were identified in PCR using primer sequences and PCR protocols as previously described (Table 1). The reaction mixture was prepared in 25 µL, which consisted of: 12.5 μL of Emerald Amp GT PCR mastermix (2 × premix) (Takara, Japan), 1 μL of each forward and reverse primer (20 pmol) (Metabion, Germany), 4.5 μL of PCR-grade water, and 6 μL of template DNA. *Salmonella* ATCC 14028 was used as positive control and included in each run. DNAse-free RNAse-free water was used as a negative control. Ten microliters of each PCR product were electrophoresed in 1.5% agarose gel with 0.5 μg/mL ethidium bromide (Sigma, Germany) in 1 × TBE buffer against a 100 bp DNA ladder (QIAGEN, USA). The gel was then visualized and photographed using a gel documentation system (Alpha Innotech, Biometra, USA).

**Table 1:** Primers sequences, annealing temperature and product size of virulence genes in *Salmonella* spp

Target gene	Primer sequence	Annealing temperature	PCR product size (bp)	References
*inv*A	5' GTGAAATTATCGCCACGTTCGGGCAA 3'	55 °C–40 s	284	[58]
	5' TCATCGCACCGTCAAAGGAACC 3'			
*sop*B	5' TCAGAAGRCGTCTAACCACTC 3'	58 °C–40 s	517	[59]
	5' TACCGTCCTCATGCACACTC 3'			
*spv*C	5' ACTCCTTGCACAACCAAATGCGGA 3'	58 °C–40 s	467	
	5' TGTCTTCTGCATTTCGCCACC 3'			
*stn*	5' TTGTGTCGCTATCACTGGCAACC 3'	59 °C–40 s	617	[60]
	5' ATTCGTAACCCGCTCTCGTCC 3'			

### Statistical analysis

Data analysis was carried out using SPSS 21.0 (IBM-SPSS Inc., Chicago, IL, USA). Categorical variables were presented as frequencies and percentages. The chi-square test was used to assess the associations between categorical variables. The threshold for statistical significance was set at P ≤ 0.05. A correlation matrix for the presence of virulence genes was performed using the “cor” function in R version (3.6.1) and visualized using the “corrplot” package. Also, the “cor.mtest” function was used to test the statistical significance of this correlation.

## Results

### Prevalence of *Salmonella* in sheep

Overall prevalence of *Salmonella* spp. in sheep was 23.3% (28/120), with 33 serotype isolates obtained (Table 2). The highest isolation rate (37.5%) was found in animals that were less than one year old followed by sheep aged between one year and two years (21.1%) and 2-year-old animals (12.9%), with higher isolation rates among ewes (29.3%) than among rams (17.7%). However, these differences were not statistically significant (P > 0.05). Diarrheic animals (85.7%, 6/7) had a significantly higher *Salmonella* isolation rate than non-diarrheic, apparently healthy ones (19.5%, 22/113) (P ˂ 0.05). Animals from Ismailia governorate showed a higher prevalence of *Salmonella* infection (28%) than those from Port-Said governorate (20%) as can be seen in Table 2.

**Table 2:** *Salmonella* infection in sheep, goats and human fecal samples

Species	No. examined	Positive cases No. (%)	No. of isolates	Age (years) Positive/total no. examined (%)			Gender		Health status		Location	
				≤ 1	1–2	≥ 2	Male	Female	Diarrheic	Non diarrheic	Ismailia	Port-Said
Sheep	120	28 (23.3%)	33^a^	12/32 (37.5%)	12/57 (21.1%)	4/31 (12.9%)	11/62 (17.7%)	17/58 (29.3%)	6/7 (85.7%)	22/113 (19.5%)	14/50 (28%)	14/70 (20%)
Goat	100	7 (7%)	7	1/19 (5.3%)	5/37 (13.5%)	1/44 (2.3%)	1/36 (2.8%)	6/64 (9.4%)	1/4 (25%)	6/96 (6.25%)	4/50 (8%)	3/50 (6%)

				≤ 10	11–20	21–30	31–40	41–50	≥ 50						
Human	100	13 (13%)	14^b^	1/33 (3.0%)	3/22 (13.6%)	2/18 (11.1%)	4/14 (28.6%)	1/7 (14.3%)	2/6 (33.3%)	6/46 (13%)	7/54 (12.9%)	3/17 (17.6%)	10/83 (12%)	6/50 (12%)	7/50 (14%)

χ^2^ for human age groups = 8.170, P = 0.147

χ^2^ for human gender = 0.000, P = 0.990

χ^2^ for sheep age groups = 5.308, P = 0.070

χ^2^ for goats age groups = 4.010, P = 0.135

χ^2^ for sheep gender = 1.621, P = 0.203

χ^2^ for goats gender = 1.540, P = 0.215

^a^Number of sheep *Salmonella* isolates is higher than number of sheep positive animals because more than one sample yielded more than one *Salmonella* serotype

^b^Number of human *Salmonella* isolates is higher than number of human positive cases because one sample yielded two different *Salmonella* serotypes

### Prevalence of *Salmonella* in goats

The prevalence of *Salmonella* infection in goat feces (7%, 7/100) was lower than that in sheep feces. Regarding the different age groups, the highest isolation rate was among goats whose ages ranged from 1 to 2 years old (13.5%) followed by animals aged less than one year (5.3%) and those aged more than two years (2.3%) (P > 0.05), as can be seen in Table 2. As was the case in sheep, the isolation rate was higher in females (9.4%) than in males (2.8%), in diarrheic goats (25%, 1/4) than in non-diarrheic ones (6.25%, 6/96), and in Ismailia (8.0%) governorate more than in Port-Said governorate (6.0%); however, these differences were not statistically significant (P > 0.05).

### Prevalence of *Salmonella* in human stool

Out of 100 human stool samples examined in the present study, 13 (13%) were positive for *Salmonella*, as can be seen in Table 2. The isolation rate in diarrheic patients (17.6%, 3/17) was higher than that in non-diarrheic ones (12%, 10/83). Furthermore, the isolation rate in Port-Said governorate (14%) was higher than that in Ismailia governorate (12%). The infection rate was similar in both genders and there was no statistically significant difference between the different age groups (P > 0.05), as can be seen in Table 2.

### Salmonella serotypes in sheep, goats, and humans

Overall, we identified 12 *Salmonella* serotypes in animals and humans (Table 3). *S*. Typhimurium (8/33, 24.2%) was the most prevalent serotype identified in sheep, followed by *S*. Enteritidis (5/33, 15.2%); however, *S.* Enteritidis was the most common serotype in goat (3/7, 42.9%) and human (4/14, 28.6%) stool samples. The least prevalent serotypes were *S*. Tsevie, *S*. Infantis (1/33, 3% each) in sheep, *S*. Apeyeme (1/7, 14.3%) in goats, and *S*. Chester (1/14, 7.14%) in humans (Table 3). Four serotypes; *S.* Typhimurium*, S.* Enteritidis*, S.* Dublin, *S.* Saintpaul, were shared between sheep, goats, and humans; moreover, both sheep and humans shared the *S*. Anatum and *S*. Essen serotypes. There was a statistically significant difference in the *Salmonella* infection rate among diarrheic and non-diarrheic sheep; however, in goats and humans, this difference was non-significant.

**Table 3:** Recovered *Salmonella* serotypes from sheep, goats and human fecal samples in relation to their health status

Recovered serotypes	Sheep^a^		Total	Goat^b^		Total	Human^c^		Total	Total No. of serotypes
	Diarrheic	Non		Diarrheic	Non		Diarrheic	Non		
*S*. Typhimurium	2 (28.6%)	8 (30.8%)	**10 (30.3%)**	–	1 (16.7%)	**1 (14.3%)**	1 (33.3%)	1 (9.1%)	**2 (14.3%)**	13
*S*. Enteritidis	1 (14.3%)	4 (15.4%)	**5 (15.2%)**	1 (100%)	2 (33.3%)	**3 (42.9)**	-	4 (36.4%)	**4 (28.6%)**	12
*S*. Heidelberg	1 (14.3%)	3 (11.5%)	**4 (12.1%)**	–	–	–	–	–	–	4
*S*. Dublin	2 (28.6%)	1 (3.8%)	**3 (9.1%)**	–	1 (16.7%)	**1 (14.3%)**	–	1 (9.1%)	**1 (7.1%)**	5
*S*. Montevideo	–	4 (15.4%)	**4 (12.1%)**	–	1 (16.7%)	**1 (14.3%)**	–	–	–	5
*S*. Saintpaul	–	3 (11.5%)	**3 (9.1%)**	–	1 (16.7%)	**1 (14.3%)**	1 (33.3%)	1 (9.1%)	**2 (14.3%)**	6
*S*. Anatum	–	1 (3.8%)	**1 (3.0%)**	–	–	–	–	2 (18.2%)	**2 (14.3%)**	3
*S*. Tsevie	–	1 (3.8%)	**1 (3.0%)**	–	–	–	–	–		1
*S*. Chester	–	–	–	–	–	–	–	1 (9.1%)	**1 (7.1%)**	1
*S*. Essen	1 (14.3%)	–	**1 (3.0%)**	–	–	–	–	1 (9.1%)	**1 (7.1%)**	2
*S*. Infantis	–	1 (3.8%)	**1 (3.0%)**	–	–	–	–	–	–	1
*S*. Apeyeme	–	–	–	–	–	–	1 (33.3%)	–	–	1
Total (%)	7/33 (21.2%)	26/33 (78.8%)		1/7 (14.2%)	6/7 (85.7%)		3/14 (21.4%)	11/14 (78.6%)		54

^a^χ^2^ = 15.365, P = 0.001

^b^χ^2^ = 2.074, P = 0.150

^c^χ^2^ = 0.391, P = 0.532

The distribution of the identified *Salmonella* serotypes in human samples in relation to their history of contact with animals is shown in Table 4. Overall, the prevalence of *Salmonella* infection in humans who have been in contact with animals including sheep and goats (28%) was significantly higher than that in people with a history of contact with animals of other species (10%) and in people with no history of contact with animals at all (7.3%) (P ˂ 0.05). Notably, 50% of the identified human *Salmonella* serotypes fall also in the class of those “with history of contact with animals including sheep and goats” that were represented in *S.* Typhimurium*, S.* Enteritidis*, S.* Dublin*, S.* Saintpaul*, S.* Chester*,* and *S.* Essen. Interestingly, four of these serotypes were the same serotypes mutually shared between sheep, goats, and humans (Table 3).

**Table 4:** Distribution of human *Salmonella* serotypes in relation to history of contact with animals

	No. examined	Positive No. (%)	Total No. of isolates	Recovered *Salmonella* serotypes No. (%)							
				*S.* Typhimurium	*S.* Enteritidis	*S.* Dublin	*S.* Saintpaul	*S.* Anatum	*S.* Chester	*S.* Essen	*S.* Apeyeme
Contact with animals including sheep and goats	25	7 (28.0)	7	1 (14.3)	2 (28.6)	1 (14.3)	1 (14.3)	1 (14.3)	1 (14.3)	–	–
Contact with animals other than sheep and goats	20	2 (10.0)	3	–	1 (33.3)	–	–	1 (33.3)	–	–	1 (33.3)
No animals contact	55	4 (7.3)	4	1 (25.0)	1 (25.0)	–	1 (25.0)	–	–	1 (25.0)	–
Total	100	13 (13.0)	14	2 (14.3)	4 (28.6)	1 (7.1)	2 (14.3)	2 (14.3)	1 (7.1)	1 (7.1)	1 (7.1)

χ^2^ = 6.728, *P* = 0.035

### Virulence determinants in *Salmonella* serotypes from sheep, goats, and humans

**Fig. 1:**
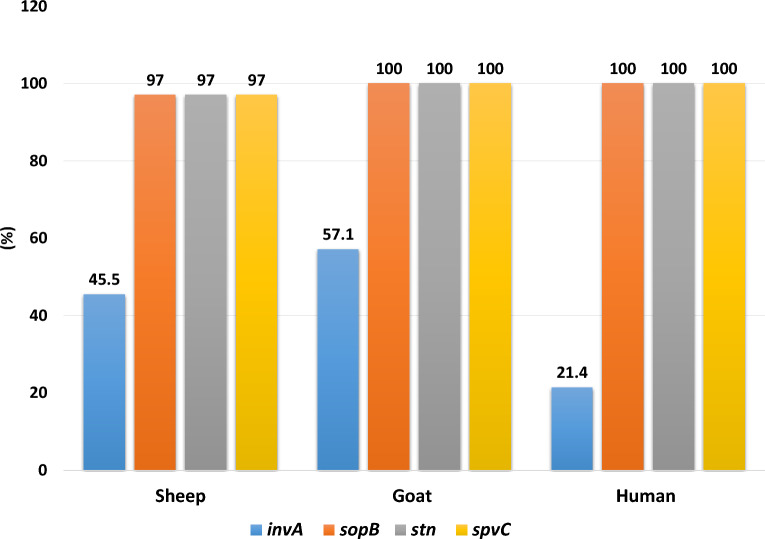
Distribution of virulence genes in fecal *Salmonella* isolates from sheep, goat, and humans

**Fig. 2:**
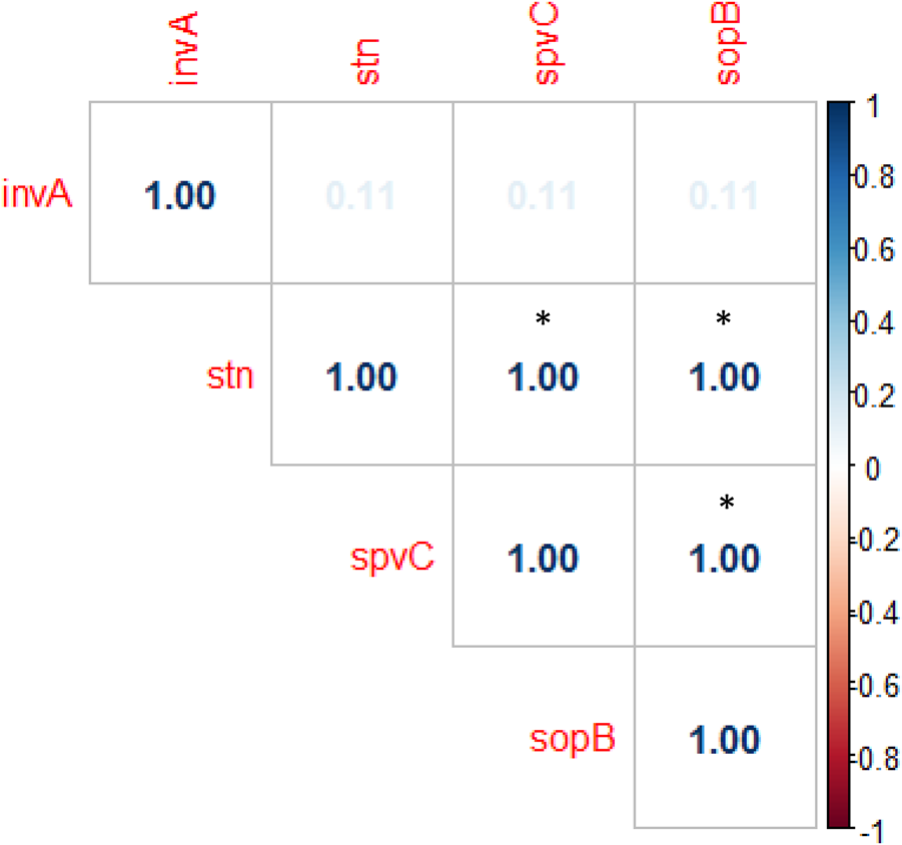
Correlation matrix for the occurrence of virulence genes in *Salmonella* serotypes. Blue color indicates positive correlation. Degree of color intensity designates the correlation value. Asterisk denotes significance of the correlation at P of 0.05

All sheep, goat, and human serotypes (except one *S*. Typhimurium isolate from sheep) successfully amplified the expected molecular sizes of *sop*B, *stn,* and *spv*C (98.1%, for each) virulence genes (Table 5). The *inv*A gene was the least frequent (22/54, 40.7%). Figure 1 shows the distribution of virulence genes in each animal species and in humans. The correlation matrix revealed that there was a significant, very strong positive correlation between the *sop*B, *stn,* and *spv*C genes (P < 0.05); however, for the *inv*A gene, there was a non-significant, very weak positive correlation with other virulence genes, as can be seen in Fig. 2.

**Table 5:** Distribution of virulence genes among total fecal *Salmonella* serotypes from animals and human

*Salmonella* serotypes	Total No. of serotypes	Virulence genes			
		*inv*A No. (%)	*sop*B No. (%)	*stn* No. (%)	*spv*C No. (%)
*S.* Typhimurium	13	8 (61.5)	12 (92.3)	12 (92.3)	12 (92.3)
*S.* Enteritidis	12	3 (25)	12 (100.0)	12 (100.0)	12 (100.0)
*S.* Heidelberg	4	0 (0.0)	4 (100.0)	4 (100.0)	4 (100.0)
*S.* Dublin	5	2 (40.0)	5 (100.0)	5 (100.0)	5 (100.0)
*S.* Montevideo	5	2 (40.0)	5 (100.0)	5 (100.0)	5 (100.0)
*S.* Saintpaul	6	3 (50.0)	6 (100.0)	6 (100.0)	6 (100.0)
*S.* Anatum	3	1 (33.3)	3 (100.0)	3 (100.0)	3 (100.0)
*S.* Tsevie	1	1 (100.0)	1 (100.0)	1 (100.0)	1 (100.0)
*S.* Chester	1	0 (0.0)	1 (100.0)	1 (100.0)	1 (100.0)
*S.* Essen	2	0 (0.0)	2 (100.0)	2 (100.0)	2 (100.0)
*S.* Apeyeme	1	1 (100.0)	1 (100.0)	1 (100.0)	1 (100.0)
*S.* Infantis	1	1 (100.0)	1 (100.0)	1 (100.0)	1 (100.0)
Total	54	22 (40.7)	53 (98.1)	53 (98.1)	53 (98.1)

## Discussion

Overall, the isolation rate of *Salmonella* from sheep fecal samples (23.3%) was significantly higher than from goats (7%) (Table 2). This was in agreement with the findings of other studies conducted in Ethiopia [16, 17] and Riyadh, Saudi Arabia [18]. In another study carried out in Egypt, the isolation rate of *Salmonella* from sheep feces (3.6%) was similar to that from goat feces (3.3%) [12]. Moreover, a higher isolation rate in goats had been recently reported in the USA (10.3%) [19]. This significant difference in the isolation rate of *Salmonella* may be attributed to several factors, including genetic differences between species, management practices, feeding behaviors, and significant regional variation [20, 21]. Additionally, the high detection rate in sheep in the present study may be due to the presence of rodents and stray dogs around sheep breeding sites. These species may amplify *Salmonella* organisms in the environment and spread them between flocks [22].

More than one sheep fecal sample yielded more than one *Salmonella* serotype (Table 2). This had also been previously observed [17], indicating that animals might be exposed to multiple sources of infection.

The differences in the *Salmonella* isolation rate from sheep of various age groups were similar to those reported in a previous study [23] in which the prevalence values of *Salmonella* infection in lambs (85.57%) from three governorates in Egypt was higher than that in adult sheep (80.41%). In another study, the prevalence of *Salmonella* was higher in adult sheep than in lambs because younger lambs were usually found dead without any symptoms [24]. In goats, the highest isolation rate of *Salmonella* was found in animals with ages that ranged from 1 year to 2 years (13.5%), as can be seen in Table 2. However, in Ethiopia, the *Salmonella* isolation rate from adult goats (100%) was higher than that from young ones (0%) [16]. This might be explained by the ability of young animals to shed *Salmonella* five times more than adult animals due to their limited immunity and the absence of stable intestinal flora in younger animals [25].

The current result concerning sheep gender was in agreement with the findings of a previous study where the *Salmonella* isolation rate from ewes (1.79%) was higher than that from rams (1.14%) in Ethiopia [16]. Higher isolation rates of *Salmonella* from female goats than from bucks in the current study were similar to findings of a previous study [25] where female goats had higher isolation rates of *Salmonella* (5.7%) than male goats (1.75%). However, in Ethiopia, the isolation rate of *Salmonella* spp. from bucks (100%) was higher than that from female goats (0%) [16]. Generally, the higher isolation rate of *Salmonella* spp. from female sheep or goats may be attributed to the exposure of female animals to additional stress factors like pregnancy and lactation, which lead to the increased shedding of the organism for prolonged durations [26]. This was also confirmed in a previous study in which the prevalence of *Salmonella* in nursing ewes was higher than that in lambs [24]. It appears that sex is an important risk factor that is significantly associated with the prevalence of *Salmonella* [25].

The higher rate of *Salmonella* isolation from diarrheic sheep (85.7%) and goats (25%) than from non-diarrheic ones (Table 2) was not surprising. This is because samples from diarrheic animals are usually expected to have higher rates of *Salmonella* infection [25]. The problem lies in the isolation of *Salmonella* from apparently healthy shedder sheep (19.5%) and goats (6.25%) in the present study, which indicates that these animals may act as a potential source of *Salmonella* infection to humans and other animals via contact. In this regard, it is worth mentioning that the ability of carrier animals to contaminate the environment by shedding *Salmonella* is increased when animals are exposed to stressors like transportation or holding before slaughter or late pregnancy [25].

Generally, these discrepancies of *Salmonella* spp. isolation rate from sheep and goats in the available literature might be due to inclusion of diseased subjects in some studies.

According to Table 3, *S*. Typhimurium and *S.* Enteritidis were the most frequent serotypes identified in sheep feces. Similar results were reported in previous Egyptian studies [12, 14]. *S*. Heidelberg which was isolated from sheep feces in the present study, has been previously isolated from human stool samples in Zimbabwe [27] and poultry workers in Egypt [28], reflecting its probable zoonotic importance. Similarly, although with different prevalence values, *S*. Montevideo (2.4%), *S*. Enteritidis (0.3%), and *S*. Anatum (0.2%) were isolated with other *Salmonella* serotypes from sheep fecal samples in the USA [24]. Moreover, *S*. Infantis which was isolated from sheep feces in the present study, had been previously isolated from human cases in Mexico [29] and in China [30], as well as, from stool samples of humans suffering from gastroenteritis in Beni-Suif province in Egypt [31].

Similar to our results from goats, *S.* Enteritidis had been isolated in previous studies from goat feces in Egypt [12, 13]. Interestingly, in later study, none of our identified serotypes was detected in their study, except two isolates of *S.* Enteritidis (0.39%) that were identified in diarrheic sheep feces in Giza governorate in Egypt [13]. Generally, variations in the distribution of the various serotypes are related to the geographic distribution of *Salmonella* serotypes from region to region, with exception of some common serotypes that maintain a high prevalence everywhere [17]. Notably, a large proportion of serotypes were identified in apparently healthy non-diarrheic sheep and goats compared to diarrheic ones (Table 3). This may be due to the *Salmonella* serovars’ host-adaptation, where strains have been shown to vary in different host ranges and virulences [32]. This reinforces their role as important sources of *Salmonella*.

Overall, the rate of occurrence of *Salmonella* spp. in human stool samples (17.6% in diarrheic and 12% in clinically healthy human subjects) in the present study were higher than that reported in other Egyptian studies; (4.4%) in patients with gastroenteritis from Minia governorate [33], (8%) from diarrheic patients in Zagazig governorate [34], and (5%) from clinically healthy dairy handlers in Assuit governorate [35]. The distribution of human *Salmonella* among different age groups (Table 2) follows the order that was previously reported [36]. In this regard, *Salmonella* was found to be the least prevalent enteric pathogen in children (1.9%) [37]. On the other hand, in the current report, there was no significant difference in the infection rate in both sexes. This was similar to the findings of previous studies where *Salmonella* microorganisms have been equally isolated from males and females [8, 38]. However, in a pooled data analysis from 8 countries, adults and elder females had significant higher incidence rates of infection than males, and this was suggested as a result of genetic and hormonal factors [39].

*S*. Enteritidis was the most prevalent serotype in human stool samples in this study. It seems that this serotype and/or *S.* Typhimurium are common nontyphoidal *Salmonella* spp. among Egyptians as reported in recent studies [40], which was also the case in Greece [41], Nigeria [42], and Florida (USA) [43]. In the present study, *S*. Apeyeme and *S*. Chester were identified in diarrheic and non-diarrheic patients, respectively, although they were not identified in animals. Interestingly, *S*. Apeyeme had been isolated from cattle feces and poultry droppings in Africa [44], from chicks in Egypt [45], as well as from poultry droppings in Vietnam [46]. Also, *S*. Chester had been isolated from the intestinal content of goats at slaughterhouses in Western Australia [47], demonstrating the probable zoonotic transmission of these serotypes to the inhabitants of rural areas.

*S.* Typhimurium and *S*. Saintpaul were identified at higher rates (33.3% each) in diarrheic stool than from non-diarrheic stool (9.1% each). On the contrary, *S*. Enteritidis, *S*. Anatum, *S*. Dublin, *S*. Chester and *S*. Essen were isolated only from non-diarrheic stool (Table 3). Similarly, *S.* Typhimurium was isolated at a high rate from diarrheic stool [27, 29], suggesting its association with the patient’s clinical condition. Also, *S*. Enteritidis (5.8%) and *S*. Anatum (8%) were detected in non-diarrheic children’s stool in Mexico [29].

*S*. Typhimurium, *S*. Enteritidis, *S*. Dublin, and *S*. Saintpaul were common serotypes between sheep, goats, and humans (Table 3). Similarly, *S*. Typhimurium and *S*. Saintpaul were found to be common serotypes between goats and humans [48]. Thus, the carriage of *Salmonella* by edible animals comes with a zoonotic potential for human infections with these microorganisms.

Contact with animals and animal products had been considered a risk factor for human infection with *Salmonella* [48]. In the present study, *S*. Typhimurium, *S*. Saintpaul, and *S*. Enteritidis were identified in people in contact with animals including sheep and goats (14.3%, 28.6%, and 14.3%) and also in people who had no contact with animals (25% each) (Table 4), which is suggestive of the probable existence of multiple sources for these serotypes, including small ruminants as reservoirs, which was further confirmed in the present study by their isolation from both sheep and goat feces.

The *Salmonella* genome encodes several virulence factors. These virulence genes are important in the invasion of the bacteria deeper into the host's tissues and their propagation inside host cells, including macrophages [49]. In the present study, there was a high prevalence of the *sop*B, *stn*, and *spv*C genes; however, the *inv*A gene was detected in 40.7% of all serotypes (Table 5; Fig. 1), suggesting that the *inv*A gene may not be suitable for the detection of *Salmonella* microorganisms. This finding was contrary to previous recommendation about its targeted use as a suitable tool for the molecular detection of *Salmonella* in animals and humans [50]. The correlation matrix revealed that the *sop*B, *stn*, and *spv*C genes were significantly positively correlated (P > 0.05, Fig. 2), suggesting that the detection of one of them is indicative of the presence of the other [51].

Similarly, the *sop*B gene had been detected in *Salmonella* isolates from sheep and goats in France (100%) [52] and in humans from Egypt [34, 53] and India [54]. Also, 50% of human *Salmonella* isolates from Egypt were found to carry *spv*C [55] and *stn* genes [34]. However, the *spv*C gene was not detected in another Egyptian study [53]. Interestingly, 31 out of 32 serotypes (26 from sheep and 6 from goats) of apparently healthy non-diarrheic sheep and goats amplified the *stn*, *spvC,* and *sopB* virulence genes, reinforcing the probable role of these silently infected animals in triggering infection in any host at any time.

In Malaysia, a lower percentage of the *stn* and *sopB* genes (50%) was detected in *S.* Typhimurium isolates from animal products, while the *invA* gene was identified in all isolates [56]. However, in Iran, there was a similar occurrence of the *sopB* gene in *S.* Enteritidis from human stool (100%), while the rates of occurrence of the *stn* and *spvC* genes were lower (92.6% and 70.4%, respectively) than the ones we found [57]. Also, the *stn* gene had been detected in all *S.* Enteritidis isolates from animal products in Malaysia [56]. On the contrary, a recent Egyptian report of 33 *S.* Heidelberg isolates from poultry and their workers revealed a higher occurrence of the *invA* gene (100%) with lower occurrences of the *stn* (72.8%), *spvC* (66.7%), and *sopB* (54.6%) genes [28].

## Conclusion

The current study revealed that apparently healthy sheep and goats are potential carriers of *Salmonella,* and they shed the organisms in their feces. Several *Salmonella* serotypes were isolated more from sheep fecal samples than from goat and human samples in the study area. Contact with sheep and goats may be a risk factor for human infection with *Salmonella*. The concomitant detection of common virulent serotypes; *S*. Typhimurium, *S*. Enteritidis, *S*. Dublin and *S*. Saintpaul, between sheep, goats, and humans is suggestive of the circulation of these serotypes between these species in the study area. The *inv*A gene could not be used alone to confirm the presence of *Salmonella*. The high occurrence of virulence genes in the identified *Salmonella* serotypes in both diarrheic and apparently healthy animals and humans necessitates continuous monitoring and reporting of *Salmonella* from other animal species in the food chain.

## Data Availability

All data generated or analyzed during this study are included in this article.
