# Correction to: Salmonella serovars in sheep and goats and their probable zoonotic potential to humans in Suez Canal Area, Egypt

**DOI:** 10.1186/s13028-024-00760-y

**Published:** 2024-09-10

**Authors:** Hanan Abd El-Halim Hawwas, Abdel-Karim Mahmoud Aboueisha, Hanaa Mohamed Fadel, Heba Sayed El‑Mahallawy

**Affiliations:** Department of Hygiene, Zoonoses and Animal Behaviour, Faculty of Veterinary Medicine, Suez Canal University, 4.5 Kilo Ring Road St, Ismailia, 41522 Egypt

**Correction to: Acta Veterinaria Scandinavica**

10.1186/s13028-022-00637-y

Following publication of the original article [1], we have been notified that reference 13 was published incorrectly.

It is now as follows:

13. Farouk MM, El-Molla A, Salib FA, Soliman YA. Epidemiology of *Salmonella* species in diarrheic sheep and goats. Pakistan J Zool. 2021;54:1–9.

It should be as follows:

13. Farouk MM, El-Molla A, Salib FA, Soliman YA. Epidemiology of *Salmonella* species in diarrheic sheep and goats. Pakistan J. Zool. 2022;54:381–9. 10.17582/journal.pjz/20201012161016.

The original article was updated.
